# Drinking and recreational water-related diseases: a bibliometric analysis (1980–2015)

**DOI:** 10.1186/s40557-016-0128-x

**Published:** 2016-09-06

**Authors:** Waleed M. Sweileh, Sa’ed H. Zyoud, Samah W. Al-Jabi, Ansam F. Sawalha, Naser Y. Shraim

**Affiliations:** 1 Department of Pharmacology/ Toxicology, College of Medicine and Health Sciences, An-Najah National University, Nablus, 44839 Palestine; 2 Department of Clinical and Community Pharmacy, College of Medicine and Health Sciences, An-Najah National University, Nablus, 44839 Palestine; 3 Department of Pharmaceutical Chemistry and Technology, College of Medicine and Health Sciences, An-Najah National University, Nablus, 44839 Palestine

**Keywords:** Drinking water, Recreational water, Disease, Bibliometrics

## Abstract

**Background:**

Water – related diseases are worldwide health concern. Microbial contamination and contaminant products in water are a source of disease outbreaks and development of cumulative toxic effects. Ensuring safe water is one of the goals to be achieved at the global level. The aim of this study was to assess publications on drinking and recreational water from a health point of view to understand current problems and future research trends in this field.

**Methods:**

Scopus, the largest scientific electronic database, was used to retrieve related articles and present the results as bibliometric tables and maps. Search query was modified manually using related terms to maximize accuracy.

**Results:**

A total of 2267 publications were retrieved with an average of 16.82 citations per article. The *h*-index of retrieved articles was 88. Visual mapping showed that *E. coli*, diarrhea, cryptosporidiosis, fluoride, arsenic, cancer, chlorine, trihalomethane, and *H. pylori* were most frequently encountered terms in title and abstract of retrieved articles. The number of articles on water microbiology was a significant (*P* < 0.01) predictor of worldwide productivity of water – related disease publications. *Journal of Water and Health* ranked first in number of publications with 136 (6.00 %) articles. The United States of America ranked first in productivity with a total of 623 (27.48 %) articles. Germany (15.44 %), India (16.00 %) and China (20.66 %) had the least international collaboration in water-related disease research. Environmental Protection Agency and Centers for Disease Prevention and Control were among top ten productive institutions. In the top ten cited articles, there were three articles about arsenic, one about aluminum, one about trihalomethane, one about nitrate, one about toxoplasmosis, one about gastroenteritis, and the remaining two articles were general ones.

**Conclusions:**

There was a linear increase in the number of publications on water – related diseases in the last decade. Arsenic, in drinking water is a serious concern. Cryptosporidiosis and other infectious gastroenteritis remain a major health risk of exposure to contaminated water. Increased number of publications from Asian countries was not associated with a high percentage of international collaboration.

## Background

According to World Health Organization (WHO), water-related diseases mainly include those due to drinking unsafe water or exposure to contaminated recreational water like swimming pools [1]. Disease outbreaks due to microbial or metal contamination of water has been reported [2–4]. Direct or indirect exposure to contaminated water has been reported to cause a wide range of health – related problems including cancer, gastrointestinal problems, dermatological problems, neuronal toxicity, birth defects, infections, and others [5–8]. Of particular concern is waterborne microbial infection and exposure to high doses of toxic metals in drinking water. *Giardia, Shigella, Salmonella*, and *Cryptosporidium, Campylobacter, Schistosoma,* and other infections have been reported due to exposure to contaminated water [8, 9]. Exposure to water contaminated with arsenic, manganese, lead, cadmium and others have been reported to be associated with many serious cardiovascular, oncology and neurology - related health problems [10–13]. Regulations and standards for drinking water safety and for safe use of recreational water has been set to minimize human health risk hazards [14–17]. One of the main goals of the United Nations Millennium Development Goals (MDG) set for 2015 was to half the proportion of people who do not have access to sustainable and safe drinking water [18]. Achievement of goals pertaining to safe drinking water requires an understanding of water-associated health problems reported from different world regions. The quantity and quality of research related to water – associated problems are indicator of the current situation of water safety in different world regions and provides an explanation of certain disease outbreaks related to unsafe water. Bibliometric analysis provides the tools to assess research trends on water-related diseases and important aspects of future research in this field with potential recommendation for international collaboration in certain topics in particular world regions like arsenic contamination of water resources in some Asian countries. Therefore, the objective of this study was to give a basic overview of research publications on water-related diseases. The lesson to be drawn from this study will be the extent of global efforts needed to be implemented in the future to eradicate water-related diseases, particularly in developing countries where water technology and resources available might not be as needed to guarantee water safety.

## Methods

In this study, Scopus database was used to retrieve articles related to drinking water or recreational water from a health point of view. The search query used in Scopus was like this:

(TITLE("drink* water*" OR "tap water" OR "ground water" OR "swimming" OR "recreational water" OR "Waterborne Disease" OR "Water disease Outbreak" AND NOT (transport OR channels OR surface OR body OR bodies OR coast* OR suppression OR complex* OR extraction OR reaction OR soluble OR emulsion OR irrigation OR remov* OR resorption OR mice OR animal OR hydration)) AND TITLE-ABS(disease OR health OR infect*)) AND PUBYEAR > 1979 AND PUBYEAR < 2016 AND (LIMIT-TO(SUBJAREA,"MEDI")) AND (LIMIT-TO (SRCTYPE,"j")) AND (EXCLUDE (DOCTYPE,"er"))

The asterisk was used to retrieve all related words. For example, the word “drink*” could retrieve both “drinking” and “drinkable” terms. The same applies for the word “water*” which could retrieve words related to water or waters. The words were used in title search to increase accuracy and minimize false positive results given that “water” is present in many non-medical articles such as chemistry, engineering and agriculture. Seven phrases related to water or water – related health terms were used in title search. The title search was followed by exclusion of all terms that are in the field of water technology or chemistry. These terms were found upon manual search of potential articles on health related articles. The search was even further sharpened by two steps, the first step was the conditional presence of the keyword “health” or “disease” or “infection” in the abstract of retrieved articles. The second step was limiting retrieved articles to all those categorized under the subject heading ‘medicine” in Scopus. To further ensure accuracy a sample of 100 highly cited articles were manually reviewed by two co-authors to ensure accuracy of the search query. Whenever the two co-authors disagreed on a certain article, a third co-author was asked to judge and decide that article. An example of a retrieved article that did not fit the scope of the search query was “Experimental study on green electrical discharge machining in tap water of Ti-6Al-4 V and parameters optimization” [19]. Whenever the search accuracy was not satisfactory due to presence of non-health water - related articles, the authors modified the search query until accuracy obtained was 100 % in the top 100 cited articles.

Analysis and graphics of data were carried out by exporting data from Scopus to Microsoft Excel and Statistical Package for Social Sciences Program (IBM SPSS Statistics for Windows, Version 22.0. Armonk, NY: IBM Corp.) For analysis and graphics. Density visualization maps and cluster analysis were carried out using VOSviewer technique (Nees Jan van Eck and Ludo Waltman, Leiden University’s Centre for Science and Technology Studies) [20]. The quality of publications was assessed using total citations, citations per article, and Hirsh-index (*h*-index). These parameters were used to assess quality of publications by journals, countries, and institutions. In addition to these parameters, impact factor (IF) was used as an indicator of journal strength publishing articles on water – related health problems. Regarding the *h*-index, it is obtained directly from Scopus. To get the *h*-index for authors, the data retrieved had to be limited to publications by each author and Scopus will calculate the total citations and *h*-index immediately as an inherent function in Scopus. Similarly, the *h*-index for a country, institution, or a journal is calculated by limiting data to the country or institution or journal that we are interested in, the Scopus will do the citation analysis and *h*-index directly. Regarding IF, it was obtained from the latest Journal Citation Report published by Thompson Reuters.

Poisson regression is a type of regression analysis that is used to test the significance of any related term as a predictor of a count variable. Poison regression requires a dependent variable and one or more independent variables as co-variate. In the current study, annual worldwide publications on water – related diseases was used as a dependent variable. Keywords used as a single independent co-variate were selected based on the keyword list produced by Scopus for the retrieved data.

## Results

### General information

**Fig. 1:**
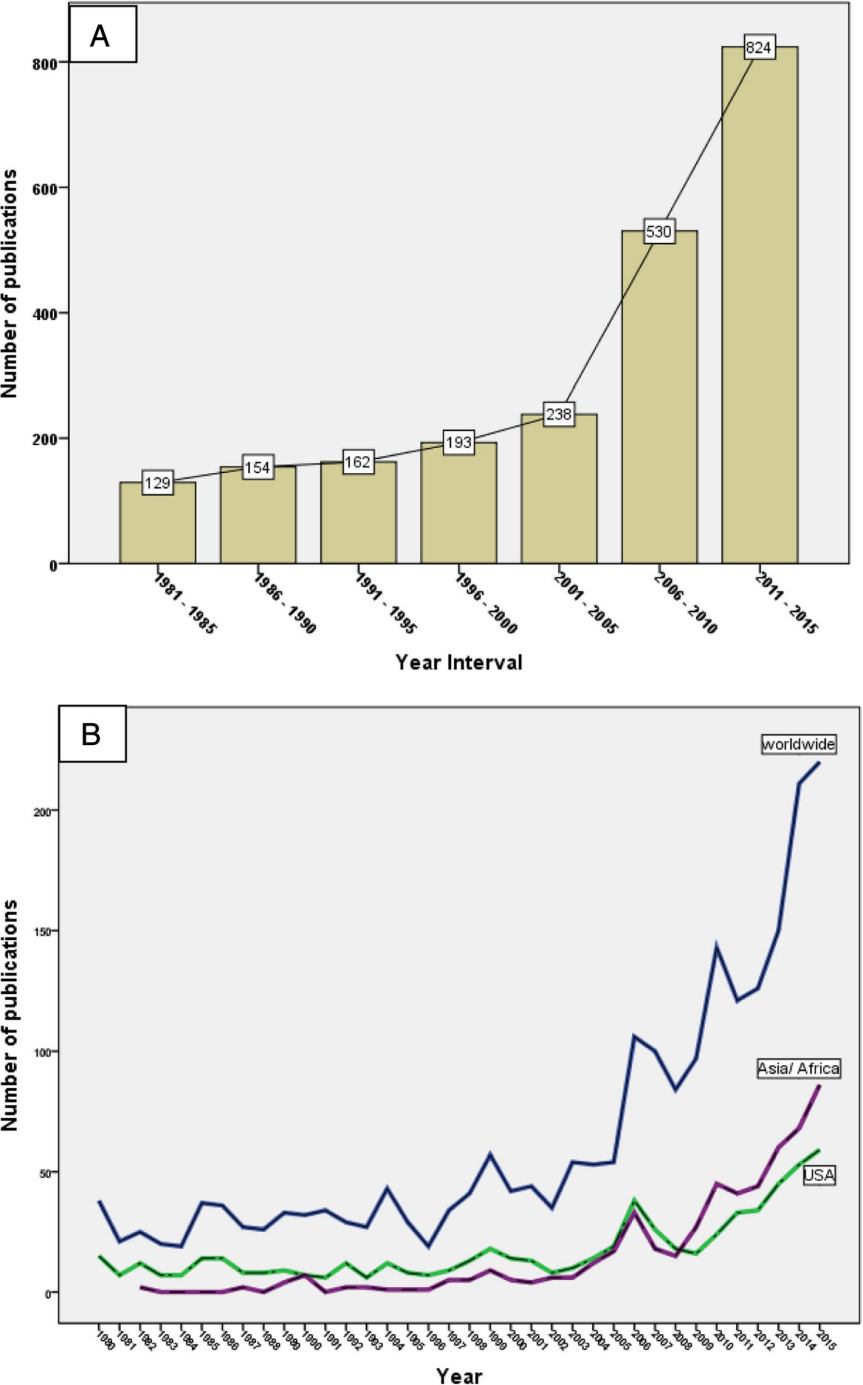
**a**: Growth of publications on water – related diseases presented as 5-year interval. The figure does not include the year 1980. **b**: Growth of publication on water-related diseases presented as worldwide versus the Unites States of America versus productivity from Asian/ African countries

**Fig. 2:**
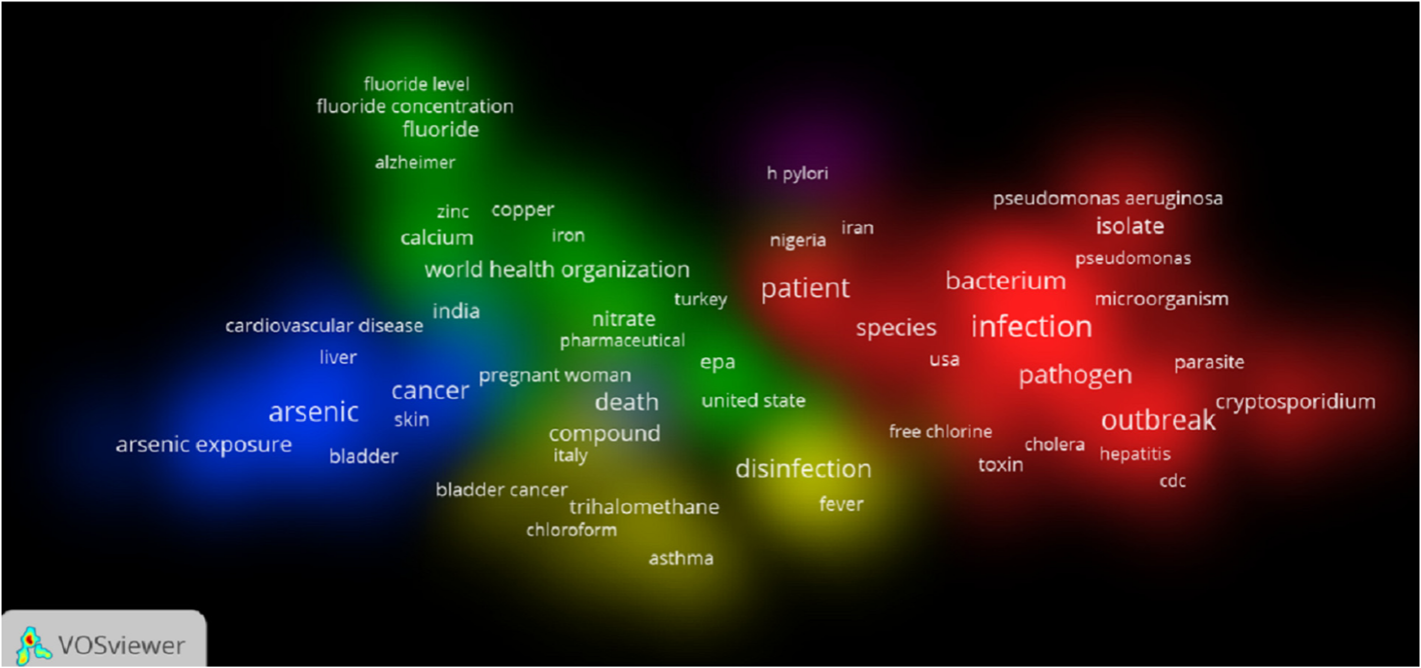
Density visualization map for frequently encountered terms in title/abstract of water related diseases publications (1980–2015)

A total of 2267 publications was retrieved from Scopus using the search query presented in the methodology section. The total citations for retrieved publications was 38,219; an average of 16.82 citations per document. The *h*-index of retrieved data was 88. The highest number of publications was recorded in 2015 with 217 publications. Fig. 1a & b show the worldwide productivity using different time scales. The number of publications was low and steady from 1980 up to 2005 followed by a stepwise increase up to 2015 (Fig. 1a). In the last decade, there were two spikes in the number of publications, one in 2006 and the other one was in 2010 (Fig. 1b). The majority of retrieved publications was original research (1936; 85.40 %). Of the total publications retrieved, 1776 (78.34 %) were written in English and the remaining articles were written in 28 different languages, mostly German (146; 6.44 %). Using VOSviewer application, the most frequent terms encountered in title/abstract of the retrieved publications were analyzed. Terms encountered at least a minimum of 10 times and pertaining to health – related conditions, contaminants, microbiology – related terms, and countries / institutions were presented. Density visualization map of 138 most frequently encountered terms is shown in Fig. 2. The map has 5 clusters. Each cluster represents closely related frequent terms. In cluster number one, the following terms were most frequent: *E. coli* (113 occurrences), diarrhea (110 occurrences), and cryptosporidiosis (82 occurrences). In cluster number two, the following terms were most common: USA (79 occurrences), EPA (77 occurrences), and fluoride (73 occurrences). Cluster number three contained the following main frequent terms: Arsenic (238 occurrences), cancer (112 occurrences), and cardiovascular (55 occurrences). In cluster number four, chlorine (62 occurrences), trihalomethane (43 occurrences), and asthma (27 occurrences) were most frequent terms. Finally, cluster number five contained one term which was *H. pylori* (31 occurrences). Other terms encountered in each cluster can be seen in the density visualization map. Of particular note is the term Bangladesh, Taiwan, Nepal which were seen in cluster number three along with arsenic. The term WHO was also seen frequently in cluster number one along with diarrhea and gastroenteritis. Applying Poisson loglinear regression and using the number of articles with keyword “microbiology” as a predictor variable showed that the number of articles on water microbiology is a significant (*P* < 0.01) predictor of worldwide productivity of water – related health publications (Table 1). The model showed that the worldwide productivity will be 1.059 times greater for each extra article published on water microbiology. In other words, there is a 5.9 % increase in the number of publications for each extra article published on water microbiology.

**Table 1:** Poisson loglinear regression for worldwide research productivity on water – related diseases using the keyword “microbiology”

Parameter	B	*P*	Exp (B)	95 % Wald Confidence Interval for Exp(B)	
				Lower	Upper
(Intercept)	3.086	.000	21.881	20.073	23.853
Keyword (microbiology)	.058	.000	1.059	1.056	1.063

Dependent variable: worldwide productivity. Predictor variable: number of articles with keyword “microbiology”. B: coefficient estimates; Exp(B): Exponentiated values of the coefficients

### Journal, country, author, and institutional productivity

The retrieved publications were published in a wide range of medical – related journals. The top ten journals involved in publishing water-related diseases are shown in Table 2. The number of different journals that published at least 10 articles on water – related diseases was 36. *Journal of Water and Health* ranked first with 136 (6.00 %) articles followed by *Environmental Health Perspectives* journal with 87 (3.84) articles. *American Journal of Epidemiology* had the highest citations per article (75.53) while *Environmental Health Perspectives* journal had the highest IF (8.440). Four of the top ten journals are issued from the United States of America (USA), two from the United Kingdom (UK), two from Germany, one from China and one from Russian Federation. The Russian and Chinese journals had lowest total citations and citations per article.

**Table 2:** Top ten journals in publishing articles on water – related diseases of water (1980–2015)

Rank	Journal	Country	No (%) N-2267	TC	C/A	*h-*index	IF
1^st^	Journal of Water and Health	UK	136 (6.00)	6745	49.60	43	1.458
2^nd^	Environmental Health Perspectives	USA	87 (3.84)	4712	54.16	38	8.440
3^rd^	Journal of Environmental Health	USA	46 (2.03)	258	5.61	9	0.963
4^th^	American Journal of Epidemiology	USA	45 (1.99)	3399	75.53	32	5.036
5^th^	Gigiena I Sanitariia	Russian Federation	43 (1.90)	7	0.16	2	N/A
6^th^	Chinese Journal Of Endemiology	China	41 (1.81)	20	0.49	2	N/A
7^th^	Health Physics	USA	35 (1.54)	542	15.49	12	1.271
8^th^	Epidemiology and Infection	UK	32 (1.41)	851	26.59	18	2.535
9^th^	Bundesgesundheitsblatt Gesundheitsforschung Gesundheitsschutz	Germany	31 (1.37)	98	3.16	6	1.499
10^th^	International Journal of Hygiene and Environmental Health	Germany	31 (1.37)	716	23.10	16	3.829

*TC* total citations

*h*-index: Hirsh index

C/A: citations per article calculated by dividing total citations by total number of publications per journal; IF: impact factor

USA ranked first in productivity with a total of 623 (27.48 %) articles (Table 3). Germany (149; 6.57 %) and the UK (141, 6.22 %) ranked second and third respectively. Half of the countries in the top ten list were European countries, two were Asians, and two were in northern America. Publications from the USA had the highest *h-*index (69) and the highest number of citations per article (30.04). Countries in the top ten list with the least international collaboration in the field of water – related diseases were Germany (15.44 %), India (16.00 %) and China (20.66 %). However, Australia (70.77 %) and the UK (46.81 %) had the highest percentage of published articles with international collaboration. Research productivity from the USA and Asian/African countries was parallel to worldwide research productivity (Fig. 1b) with a significant correlation (*p* < 0.01, *r* = 0.99).

**Table 3:** Top ten countries in publishing articles on water – related diseases (1980–2015)

Rank	Country	Frequency (%) *N* = 2267	TC	*h-*index	C/A	Number Of collaborating countries	SCP (%)	MCP (%)
1^st^	United States	623 (27.48)	18713	69	30.04	78	439 (70.47)	184 (29.53)
2^nd^	Germany	149 (6.57)	1811	23	12.15	39	126 (84.56)	23 (15.44)
3^rd^	United Kingdom	141 (6.22)	3391	32	24.05	39	75 (53.19)	66 (46.81)
4^th^	Canada	126 (5.56)	3580	30	28.41	21	85 (67.46)	41 (32.54)
5^th^	China	121 (5.34)	554	12	4.58	16	96 (79.34)	25 (20.66)
6^th^	India	75 (3.31)	922	16	12.29	6	63 (84.00)	12 (16.00)
7^th^	France	74 (3.26)	1319	19	17.82	39	51 (68.92)	23 (31.08)
8^th^	Australia	65 (2.87)	1344	19	20.68	21	19 (29.23)	46 (70.77)
9^th^	Italy	64 (2.82)	660	16	10.31	32	49 (76.56)	15 (23.44)
10^th^	Spain	61 (2.69)	1071	16	17.56	32	38 (62.30)	23 (37.70)

*TC* total citations

*h*-index: Hirsh index

C/A: citations per article calculated by dividing total citations by total number of publications per journal

*SCP* single country publication. The percentage is calculated by dividing the number of SCP by number of publications retrieved for that particular country

*MCP* multiple country publication. The percentage was calculated by dividing the total number of MCP by total number of publications retrieved for that particular country

Regarding productivity from institutions, the Environmental Protection Agency (EPA) and Centers for Disease Prevention and Control (CDC) ranked first and second respectively (Table 4). Six of the top ten productive institutions were based in the USA, one was WHO, while the remaining three were based in the UK, Germany and Taiwan. There was a strong significant and inverse relationship (*r* = - 0.83, *p* < 0.01) between rank of the institution and the total citations for publication for each institution. Institution which ranked first had the highest total citation while those in rank number ten had the least total citations. Similar relationship existed between rank of the institution and the h-index (*r* = - 0.913, *p* < 0.01).

**Table 4:** Top ten institutions in publishing articles on water – related diseases (1980–2015)

Rank^a^	Institution	Country	Frequency (%) *N* = 2267	TC	*h-*index
1^st^	United States Environmental Protection Agency	USA	84 (3.71)	3076	28
2^nd^	Centers for Disease Control and Prevention	USA	82 (3.62)	2695	28
3^rd^	The University of North Carolina at Chapel Hill	USA	38 (1.68)	1288	18
4^th^	UC Berkeley	USA	33 (1.46)	2969	20
5^th^	Columbia University Medical Center	USA	23 (1.01)	982	14
6^th^	Harvard School of Public Health	USA	20 (0.88)	878	13
7^th^	Organisation Mondiale de la Sante	WHO	19 (0.84)	512	11
7^th^	London School of Hygiene & Tropical Medicine	UK	19 (0.84)	267	9
9^th^	Umweltbundesamt, Germany	Germany	17 (0.75)	552	9
10^th^	Kaohsiung Medical University	Taiwan	15 (0.66)	645	11

*TC* total citations

*h*-index:Hirsh index

^a^Institutes having similar number of publications were given the same ranking number, and then a gap is left in the ranking numbers

Regarding top productive authors, no significant dominance was seen and most authors in the top ten list had research productivity between 14–22 articles (Table 5). However, the majority of authors (90 %) in the top ten list were from the USA while the last one in the list was from Spain. Top ten productive authors is shown in Table 5. There was no significant correlation (*p* > 0.05) between the rank of the author and the percentage of highly cited articles published by the authors.

**Table 5:** Top ten productive authors on water-related disease as retrieved based on search query used (1980–2015)

Rank^a^	Author	No (%) *N* = 2267	Country ^b^	Cluster # in map	Number of articles with ≥50 citations
1^st^	Beach, M.J.	22 (0.97)	CDC/ USA	3	9 (40.91)
2^nd^	Craun, G.F.	21 (0.93)	USA	3	11 (52.38)
3^rd^	Wade, T.J.	19 (0.84)	EPA/ USA	3	5 (26.32)
4^th^	Calderon, R.L.	17 (0.75)	EPA/ USA	3	11 (64.71)
5^th^	Ahsan, H.	16 (0.71)	USA	1	6 (37.50)
5^th^	Parvez, F.	16 (0.71)	USA	1	5 (31.25)
7^th^	Colford, J.M.	15 (0.66)	USA	2	3 (20.00)
8^th^	Chen, Y.	14 (0.62)	USA	1	5 (35.71)
8^th^	Smith, A.H.	14 (0.62)	USA	out	9 (64.29)
8^th^	Villanueva, C.M.	14 (0.62)	Spain	out	4 (28.57)

^a^Authors having similar number of publications were given the same ranking number, and then a gap is left in the ranking numbers

^b^Country affiliation for each author was extracted from Scopus at the date of analysis (July 13^th^, 2016)

### Citation analysis and most cited articles

**Fig. 3:**
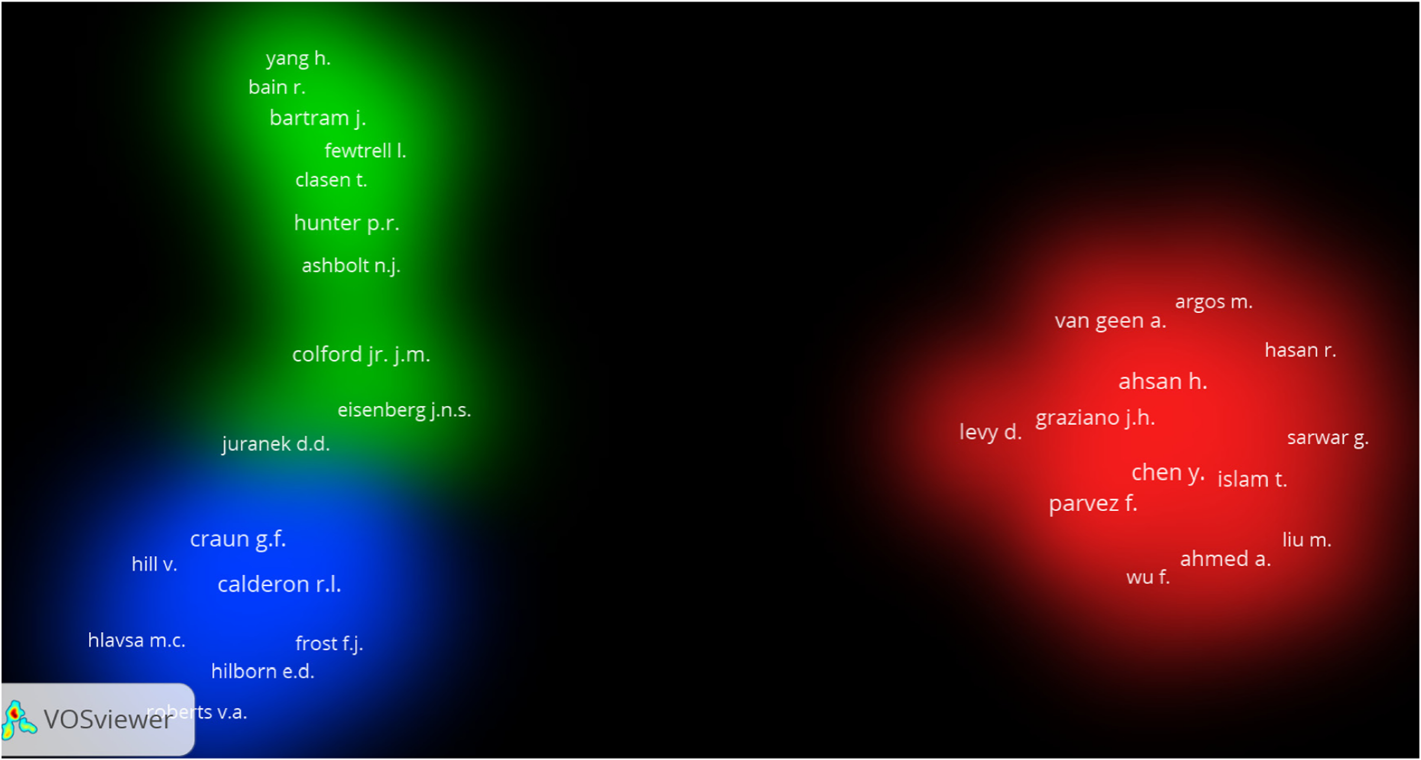
Density visualization map of co-author analysis of water related diseases publications (1980–2015). Some names might not be seen due to overlap of names or limited magnification power

A total of 1702 (75.07 %) articles were cited at least once; the remaining articles were not cited at all. Cited articles were further analyzed using VOSviewer to create visualization maps. Co-authorship analysis using VOSviewer showed three clusters of authors (Fig. 3). Cluster number one included 14 authors, three of them were among the top ten productive authors: Parvez, F (116 co-authorships), Ahsan, H (113 co-authorships), and Chen, Y (112 co-authorships). Authors with higher number of co-authorships had higher collaboration compared with those with lower number of co-authorships. Furthermore, authors in the same cluster are those with closer collaboration compared to authors who exist in other clusters. Cluster number two included 12 authors, one of them was from the top ten productive authors; Colford Jr, J.M (17 co-authorships). Cluster number three included 11 authors, four of them were in the top ten productive list: Wade, T.J (47 co-authorships), Calderon, R.I (34 co-authorships), Craun, G.F (36 co-authorships), and Beach M.J (36 co-authorships).

The top cited articles are shown in Table 6 [6, 7, 9, 21–27]. The top cited article was about arsenic in drinking water in Bangladesh and received a total of 919 citations. The article was published in the *Bulletin of the World Health Organization* journal. The second ranked article in number of citations was also about arsenic in drinking water and its association with cancer in North Chile. The article received a total of 495 citations and was published in *American Journal of Epidemiology*. The two articles in the first and second rank in number of citations were published by the same author group and were about arsenic in drinking water. A third article on arsenic was in rank 7^th^ and was about association between arsenic in drinking water and internal cancer. Of the top ten cited articles, there were about arsenic, one was about aluminum and its association with Alzheimer’s disease, one was about association between trihalomethane in drinking water and spontaneous abortion, one was about acceptable levels of nitrate in drinking water, one was about toxoplasmosis infection due to exposure to contaminated water, one was about gastroenteritis due to exposure to contaminated recreational water, and the remaining two articles were about contaminated water and its general health effects.

**Table 6:** Top ten cited articles on water-related diseases (1980–2015) [6, 7, 9, 21–27]

Rank	Authors	Title	Year	Source title	Number of citations
1^st^	Smith et al [26]	Contamination of drinking-water by arsenic in Bangladesh: A public health emergency	2000	Bulletin of the World Health Organization	919
2^nd^	Smith et al [25]	Marked increase in bladder and lung cancer mortality in a region of northern Chile due to arsenic in drinking water	1998	American Journal of Epidemiology	495
3^rd^	Curriero et al [22]	The association between extreme precipitation and waterborne disease outbreaks in the United States, 1948–1994	2001	American Journal of Public Health	425
4^th^	Martyn et al [23]	Geographical relation between Alzheimer’s disease and aluminium in drinking water	1989	Lancet	403
5^th^	Bowie et al [9]	Outbreak of toxoplasmosis associated with municipal drinking water	1997	Lancet	366
6^th^	Cabelli et al [21]	Swimming-associated gastroenteritis and water quality	1982	American Journal of Epidemiology	279
7^th^	Morales et al [6]	Risk of internal cancers from arsenic in drinking water	2000	Environmental Health Perspectives	278
8^th^	Prüss [24]	Review of epidemiological studies on health effects from exposure to recreational water	1998	International Journal of Epidemiology	275
9^th^	Ward et al [27]	Workgroup report: Drinking-water nitrate and health - Recent findings and research needs	2005	Environmental Health Perspectives	268
10^th^	Waller et al [7]	Trihalomethanes in drinking water and spontaneous abortion	1998	Epidemiology	267

## Discussion

In this manuscript, we tried to present a bibliometric overview of water-related publications on health-related diseases that includes a wide range of possible infections due to microbial contaminations of water or toxicities associated with cancer or cardiovascular or neuronal disorders due to exposure to materials like heavy metals present in drinking water. Several bibliometric analyses on water publications have been carried out that focused on one aspect like arsenic or lead in drinking water or infection with cryptosporidium [28–30]. However, no studies have been carried out to assess the overall health aspects of unsafe drinking or recreational water. Bibliometric analysis on water in general and water technologies have been also carried out without focusing on health related issues [31–34].

Our study showed that there is a growing interest and research activity on this topic manifested as an increasing trend in the number of publications particularly in the last decade. Furthermore, this interest is being witnessed in different parts of the world manifested in the diversity of geographical distribution of countries in the top ten list. No doubt that governmental and non-governmental international health bodies like WHO, CDC and EPA are taking the lead in this topic manifested in top ten productive institutions and authors. Finally, the manuscript showed that microbial and toxic related health issues are being heavily addressed. The toxic effects of water contaminants and microbial contamination are being a serious concern particularly in developing countries while risk of infections and negative health effects of recreational and swimming pools are being a concern in developed countries [35–38]. It has been reported that diarrheal disease mostly due to contaminated drinking water accounts for 4.1 % of the total Disability Adjusted Life Year (DALY) global burden of disease and is responsible for the deaths of 2 million people every year [39]. The high h-index of the retrieved publications is a strong indicator of the value and importance of such publications. The importance of water-related diseases was emphasized by dedicating a World Water Day (March 22, 2013) [40]. The top cited articles reveal the hot topics in water-related health research and topics that are of real concern to health organization and health policymakers. Health issues like arsenic and cancer, aluminum and Alzheimer’s disease, trihalomethane toxicity in drinking water, risk of gastroenteritis from recreational water, disease outbreaks, and Bangladesh as a country at high risk of arsenic toxicity in drinking water.

Journals encountered in the top ten productive list were mainly in the field of environmental health or epidemiology. Specialized journal in water was also present and ranked first among top ten journals publishing on water-related diseases. The subject of water and health is a multidisciplinary one and therefore various types of journals were encountered includes ones in the field of chemistry, environment, epidemiology, infection, toxicology, public health and others. That is one reason why no significant dominance was seen among different types of journals excluding the specialized journal of *Journal of Water and Health*. The findings that Chinese and Russian journals had the least citations and h-index when compared with other journals in the top ten list could be due to the language of publication where English remains the scientific language for researches. Despite that, the findings that a Russian and a Chinese journal were among the top ten list of journals is indicative of how common water – related health problems are in all world regions.

Countries included in the top ten productive list were also diverse and included a bulk of European countries, northern American countries, Asian countries and Australia. However, countries from regions like Africa or Latin America were missing from the top ten list. For the USA to occupy the first rank in the list was not surprising given the research facilities and funds available for health – related projects in the USA. Furthermore, the EPA and CDC are being actively involved in water – related health issues and that is why both EPA and CDC occupied top ranks in top productive institutions. Bibliometric studies in other medical fields also showed dominance of the USA over other countries in number and quality of publications in many medical fields. In the current manuscript the productivity of the USA was more than one fourth of worldwide research productivity. The USA has witnessed several waterborne related disease outbreaks (WBRDOs). The CDC reported that a total of 32 outbreaks of water-related diseases in 2011 or 2012 and resulted in at least 431 cases, 102 hospitalizations (24 % of cases), and 14 deaths [3]. Another study indicated that from 1971 to 2006, a total of 833 waterborne disease outbreaks with 577,991 cases of illness, and 106 deaths were reported [41]. Microbial contamination such as cryptosporidiosis, *E. coli*, Norovirus, *Legionella*, *Giardia* and other infectious agents seem to be the leading cause of WBRDOs in the USA and other developing countries [41–44]. The top ten productive institutions included many academic and research centers in the USA. However, two institutions/ organizations in the top ten list is worth commenting, the WHO and the *Kaohsiung Medical University*in Taiwan. The WHO has published a series of reports on water quality, technology and water related-diseases. The WHO is being involved in developing preventive strategies for water-related diseases [45]. Furthermore, the WHO is involved in estimating national burden of water-related diseases and national guidelines for good water quality [46, 47]. The *Kaohsiung Medical University* in Taiwan is being listed as one of the top ten productive institutions in water-related diseases worldwide. Some of the publications of this academic center has focused on the role of arsenic and other heavy metals in drinking water and its association with cancer [48–51].

Our study has few limitations that need to be mentioned. Most of these limitations are similar to the ones listed in previous bibliometric studies and they are inherent to the technique itself and database chosen to retrieve data [52–55]. It is important to remember that Scopus retrieve article with English title and abstract regardless of the language of the manuscript. Therefore, articles written completely in non-English language were not retrieved. For example, articles published in Persian language in a journal not indexed in Scopus will not be retrieved. It is the policy of Scopus to have all articles of journals indexed in Scopus to have an English title and abstract. This might have created some bias in data. However, articles with no English title or abstract are of local interest and mostly of little international impact compared with those having English title and abstract and therefore readable by researchers all over the world. In retrieving articles, the search query was built by authors based on literature and on manual review of retrieved articles. Therefore, the results in this study remain valid within the context of search query which was confined by title search and by category of journals under the subject “medicine” in Scopus. Finally, the citation analysis presented in this manuscript did not exclude self-citation which is common in literature and might not be a point of strength for authors and journals. All authors listed in this manuscript were presented as retrieved from Scopus and based on the data present in retrieved articles. The authors of this manuscript did their best to avoid false positive and false negative results by manual review of hundreds of retrieved articles. Furthermore, the authors tried their best to confine data to articles related to water-related health problems and exclude articles in pure chemistry, engineering, technology, and physics.

## Conclusions

This study showed that there was a noticeable and almost linear increase in the number of drinking and recreational water health related publications. Major contribution of these publications came from the USA and Europe. Institutions and international health centers like EPA, CDC, and WHO are taking a prominent role in this filed. Arsenic, other heavy metals, gastroenteritis and cryptosporidiosis are important health – related problems encountered in drinking and recreational water. Research productivity on water – related diseases from Asia and Africa have witnessed an upward increase in the last few years. However, research from Asian countries like India and China was characterized with low percentage of international collaboration. The number of studies linking arsenic and other heavy metals to various types of cancer require global action particularly in countries such as Bangladesh.

## Abbreviations

EPA: Environmental Protection Agency
CDC: Centers for Disease Prevention and Control
WHO: World Health Organization
WBRDOBs: Waterborne related disease outbreaks
